# Selfing ability and drift load evolve with range expansion

**DOI:** 10.1002/evl3.136

**Published:** 2019-08-29

**Authors:** Matthew H. Koski, Nathan C. Layman, Carly J. Prior, Jeremiah W. Busch, Laura F. Galloway

**Affiliations:** ^1^ Department of Biology University of Virginia Charlottesville Virginia 22902; ^2^ Current Address: Department of Biological Sciences Clemson University Clemson SC 29631; ^3^ School of Biological Sciences Washington State University Pullman Washington 99164

**Keywords:** Baker's rule, bottleneck, expansion load, genetic drift, heterosis, inbreeding depression, mating system, postglacial range expansion

## Abstract

Colonization at expanding range edges often involves few founders, reducing effective population size. This process can promote the evolution of self‐fertilization, but implicating historical processes as drivers of trait evolution is often difficult and requires an explicit model of biogeographic history. In plants, contemporary limits to outcrossing are often invoked as evolutionary drivers of self‐fertilization, but historical expansions may shape mating system diversity, with leading‐edge populations evolving elevated selfing ability. In a widespread plant, *Campanula americana*, we identified a glacial refugium in the southern Appalachian Mountains from spatial patterns of genetic drift among 24 populations. Populations farther from this refugium have smaller effective sizes and fewer rare alleles. They also displayed elevated heterosis in among‐population crosses, reflecting the accumulation of deleterious mutations during range expansion. Although populations with elevated heterosis had reduced segregating mutation load, the magnitude of inbreeding depression lacked geographic pattern. The ability to self‐fertilize was strongly positively correlated with the distance from the refugium and mutation accumulation—a pattern that contrasts sharply with contemporary mate and pollinator limitation. In this and other species, diversity in sexual systems may reflect the legacy of evolution in small, colonizing populations, with little or no relation to the ecology of modern populations.

**Impact Summary**

Following the last glacial recession, many species expanded their ranges from source populations in regions of suitable habitat called glacial refugia. These range expansions have left lasting imprints on the evolution and genetic diversity of populations. Mating systems are highly variable among hermaphroditic organisms ranging from obligate outcrossing to nearly complete selfing, and their evolution is predicted to be strongly impacted by range expansion. Although considerable effort has been made to explain this variation in light of selection in recent time, there is substantial variation not accounted by this explanation. Here, we evaluate the extent to which patterns of historical colonization have shaped mating system diversity by correlating variation in autonomous self‐fertilization with genetic signatures of postglacial range expansion. In the widespread herb American Bellflower, we detected high levels of neutral genomic variation in a putative glacial refugial region and a reduction in diversity outside of this region, reflecting population bottlenecks. Populations farthest from the refugium expressed high genetic load, suggesting that colonization events facilitated the accumulation of deleterious mutations. The ability to self‐fertilize was positively correlated with distance from the refugium and with the amount of genetic drift, linking variation in a key mating system trait to a history of range expansion. Although range expansion has been shown to structure the distribution of genetic variation, we show that it also affects the evolution of reproductive traits. Historical range expansion from glacial refugia is pervasive and has the potential to shape mating system diversity across many organisms. Furthermore, mating system evolution will likely accompany future poleward range shifts expected to occur under globally changing climates.

Contemporary species distributions are strongly influenced by past range expansions and contractions. In temperate regions across the globe, range expansion following glacial recession has shaped population genetic diversity and structure (Hewitt 2000; Davis & Shaw 2001; Petit 2003; Schonswetter et al. 2005; Barnard‐Kubow et al. 2015). For example, in the Northern Hemisphere, genetic variation often declines at the more northerly reaches of species’ distributions (Alexandrino et al. 2000; Comps et al. 2001; Widmer & Lexer 2001; Obbard et al. 2006; Griffin & Willi 2014). A similar pattern of genetic diversity among disparately related taxa has likely arisen because colonization of new environments often involves few individuals, leading to serial population bottlenecks through space and time. Repeated founder events during each successive stage of range expansion is expected to reduce diversity throughout the genome (Nei et al. 1975), with the potential to strongly influence the viability of natural populations and the trajectory of trait evolution (Peischl & Excoffier 2015; Henn et al. 2016).

Although any trait may evolve during geographic range expansion, mating systems are expected to be particularly sensitive to a history of colonization (Baker 1955; Hargreaves & Eckert 2014; Pannell et al. 2015). Two primary processes can shape patterns of mating system diversity along routes of range expansion in flowering plants. First, selection may favor self‐fertilization if mates or pollinators limit seed production (Schoen et al. 1996; Morgan & Wilson 2005), as would be expected if there are few colonists at an expanding wave front (Grossenbacher et al. 2015, 2017). Second, reduced fitness of inbred progeny (i.e., inbreeding depression), a primary deterrent to the evolution of selfing (Fisher 1941; Layman et al. 2017), is expected to reflect recent demographic history. Specifically, the magnitude of inbreeding depression caused by segregating deleterious alleles, (i.e., segregating load), is expected to decline with the reduction in genetic diversity in populations that have experienced bottlenecks (Kirkpatrick & Jarne 2000), with greater declines in populations that are effectively small for long periods of time (Bataillon & Kirkpatrick 2000). Reduced inbreeding depression may result in more permissive conditions for the evolution of self‐fertilization (Pujol et al. 2009). Indeed, reduced inbreeding depression has been documented at range edges and in populations farthest from sources in some taxa (Pujol et al. 2009; Barringer et al. 2012), but whether this is associated with the evolution of elevated self‐fertilization is not known.

Recurrent founder events during colonization should reduce effective population size (Wade & McCauley 1988; Peter & Slatkin 2013), particularly if successful founders are drawn from locations near the wave front (Hallatschek & Nelson 2008). In these cases, an accumulation of deleterious mutations known as the expansion load (or drift load) may occur (Peischl et al. 2013; Henn et al. 2016; Peischl et al. 2018). Crosses between populations with high expansion load should yield heterosis or hybrid vigor, particularly for those with the smallest effective sizes (Whitlock et al. 2000; Oakley et al. 2015; Spigler et al. 2017; Willi et al. 2018). Evolution of the mating system has been associated with reductions in effective population size (Busch et al. 2011; Willi et al. 2018), which is supported by the robust association between island colonization and self‐fertility (Pannell et al. 2015; Grossenbacher et al. 2017). However, most studies of variation in mating systems explore its association with reproductive assurance in contemporary environments (Eckert 2002; Herlihy & Eckert 2005; Koski et al. 2017; Opedal et al. 2016). Mixed support for this hypothesis suggests that a historical perspective may be crucial for explaining diversity in mating system traits. However, it is often challenging to implicate historical processes in driving evolution (Brandvain et al. 2013).

The self‐compatible North American herb *Campanula americana* is an ideal model for contrasting contemporary and historical processes in predicting the evolution of selfing traits. Importantly, there is clinal variation among populations in the capacity to autonomously self‐fertilize—populations in the northwestern part of the range self‐fertilize more readily than those in the southeast (Koski et al. 2017). Koski et al. (2017) found variation among natural populations in mate and pollen limitation across two years. However this variation was not associated with the geographic cline in autonomy, suggesting that contemporary ecological selective conditions are unlikely to underlie variation in this important mating system trait. Furthermore, phylogeographic patterns suggest this species persisted in a putative glacial refugium in the southeastern United States during the Pleistocene glaciation and recently expanded its geographic range (Barnard‐Kubow et al. 2015). Here, we test whether this historical biogeography explains variation among populations in the ability to self‐fertilize.

If historically small populations present the most conducive opportunities for the evolution of autonomy, this trait should be strongly associated with signatures of genetic drift (Schoen et al. 1996; Barrett et al. 2014). Such signatures would be expected in populations that have undergone repeated population bottlenecks during a geographic range expansion. We ask four questions to evaluate the possible evolution of autonomy in historically small populations: (1) Given footprints of genetic drift on patterns of genetic diversity, where is the most likely location of a glacial refugium in *C. americana*? (2) Does the load of fixed or locally common mutations (i.e., drift load) increase with distance from the refugium? (3) Does the segregating genetic load (i.e., inbreeding depression) decline with distance from the refugium? and (4) Do populations more distant from the refugium display an elevated capacity for autonomous selfing, and is this associated with fixed and segregating load?

## Methods

### SYSTEM

The American bellflower, *C. americana* L. (*= Campanulastrum americanum* Small), is a widespread, autotetraploid herb (Galloway et al. 2003). This species is native to Eastern North America and its showy protandrous flowers are bee pollinated. Although largely outcrossing in natural populations (Galloway et al. 2003; Koski et al. 2019), its mating system is flexible depending on the pollination environment (Leibman et al. 2018). Populations vary substantially in their ability to produce autonomously self‐fertilized fruits because of variation in the overlap of male and female sexual functions, and the timing of pollen germinability (Koski et al. 2018). The capacity to autonomously self is higher in populations in the northwestern part of the range (e.g., Minnesota and Iowa) (Koski et al. 2017). Here, we work with 24 previously described populations west of the Appalachian Mountains (Koski et al. 2017; Table S1).

In *C. americana*, analyses of the chloroplast and nuclear genome identified three main clades. The most divergent includes populations located in the Appalachian Mountains (Barnard‐Kubow et al. 2015). The two remaining clades include a monophyletic group composed of all populations west of the Appalachian Mountains (focal clade) and another group of populations in the Atlantic Coast and the Smoky Mountains, a subrange of the Appalachian Mountains. Although the location of ancestors for the clade west of the Appalachians is unknown, habitats in the Gulf Coast, Atlantic Coast, and southern Appalachian Mountains are the most likely candidates (Barnard‐Kubow et al. 2015). In this study, we use populations from the Appalachians, Atlantic Coast, and Gulf Coast as outgroups to the focal western clade.

### AUTONOMOUS FRUIT SET

We scored autonomous fruit set as described in Koski et al. (2017). Briefly, on greenhouse‐grown plants from each population, two flowering nodes were marked. After plants set fruit in the absence of pollinators, we tallied the number of flowers and fruits produced at each node, which are persistent. We divided the number of fruits by the number of flowers as a measure of autonomous fruit set for each plant and calculated an average for each population.

### POPULATION GENETICS

#### Population sampling, DNA sequencing, and population genomic diversity

Plants were grown from seed in a greenhouse and leaf tissue was collected from seven plants in 24 focal populations (Table S1). An additional 10 plants from outgroup populations were sampled. Genomic DNA was extracted with a modified CTAB protocol. The quantity of individual double‐stranded DNA was assessed using a Qbit 4 fluorometer manufactured by Life Technologies Holdings Pte Ltd, Singapore (Invitrogen, Inc.). Library preparation was conducted by Floragenex, Inc (Portland, OR, USA). Briefly, samples were normalized to 20 ng/µL, treated with RNase A, and digested with *SbfI* restriction endonuclease. Unique 10 bp barcodes and adaptors were ligated to fragments, which were pooled and sheared with a Bioruptor NGS sonicator (Diagenode, Inc.). Fragments were size selected using gel extraction (300–500 bp range) and amplified by PCR using established protocols (Puebla et al. 2014). These amplicons were subjected to paired‐end sequencing using an Illumina HISeq 2500 (Illumina, Inc.) at the University of Oregon Genomics Core Facility.

Sequence data were processed in ipyrad (Eaton 2014). Paired‐end reads (2 × 101 bp) were aligned to a shotgun‐sequence of *C. americana* (K. Barnard‐Kubow, unpubl. data). Low‐quality samples were removed, using parameters defined in the available ipyrad parameter file. A minimum of 20 reads were required for each plant at nucleotide sites, and alleles below a frequency of 5% were removed from populations. Contigs absent in six or more populations were removed, as were those with a length below 50 bp. After filtering, populations had a mean of 68.72 kb of nucleotides (Table 1). The mutation parameter was estimated using Watterson's (θ_W_) and Tajima's estimators (θ_π_), in addition to Tajima's *D* (Tajima 1989; Arnold et al. 2012). In populations at demographic equilibrium, Tajima's *D* values are expected to be near zero. The sensitivity of θ_W_ to recent declines or increases in population size, however, generates positive or negative Tajima's *D* values, respectively, with re‐equilibration expected over time (Tajima 1989).

**Table 1:** The geography of population genomic diversity of *Campanula americana*

Population	Distance from refugium (km)	Total bp	$\theta_{W}$ (× 10^3^)	$\theta_{\pi }$ (× 10^3^)	Tajima's *D*
KY51	121	68,877	9.697	9.493	−0.032
OH119	322	70,619	9.968	10.585	0.136
IN77	331	69,169	6.992	8.020	0.249
IN46	347	71,996	8.174	8.668	0.078
ALBG	378	68,256	7.855	8.984	0.223
TN19	432	67,433	6.990	8.062	0.263
OH64	487	68,633	7.628	8.447	0.174
PA27	534	66,719	5.622	6.696	0.285
AL2012	574	66,657	6.822	7.903	0.198
MI127	606	70,537	6.563	7.738	0.281
MI126	611	68,317	5.730	6.982	0.251
AL79	625	64,240	7.991	8.776	0.167
MO49	687	70,488	8.818	9.570	0.157
MO116	691	71,798	7.344	7.941	0.132
AR56	709	69,202	7.186	7.899	0.146
MO57	775	66,835	7.337	7.816	0.143
MO115	775	71,041	7.712	8.259	0.117
AR125	831	68,335	6.837	7.938	0.282
WI128	880	66,230	4.191	5.293	0.283
IA10	1037	66,563	6.346	7.534	0.297
OK61	1058	67,494	4.830	5.936	0.308
KS60	1075	69,821	7.020	8.095	0.221
MN117	1195	69,150	6.801	7.587	0.192
MN118	1366	70,914	6.210	7.342	0.250

Mutation parameters were estimated from segregating sites $(\theta_{W})\;$or pairwise nucleotide differences $\left(\theta_{\pi }\right)$.

If plants produce offspring through partial self‐fertilization, elevated homozygosity reduces effective population size (Schoen et al. 1996). Such declines are proportional to the inbreeding coefficient (*F*; Charlesworth & Wright 2001). Expected heterozygosity equaled gene diversity, and observed heterozygosity followed from the gametic heterozygosity of each genotype (Moody et al. 1993). The inbreeding coefficient was calculated as the proportional decline in heterozygosity, 1 − *H*_obs_/*H*_exp_ (Meirmans et al. 2018). We tested the hypothesis that higher autonomy increases homozygosity within populations by correlating population‐level autogamy and inbreeding coefficients.

#### Identifying the refugium as the origin of a geographic range expansion

Reductions in population size associated with the colonization of new environments decrease effective population size (*N_e_*), with serial founding events leading to more pronounced declines. Because random sampling associated with genetic drift causes a deficit of mid‐frequency variants, the site frequency spectrum is expected to systematically shift to more extreme values as populations decline in their effective size (Crow & Kimura 1970). When the loss of derived alleles is ignored, enhanced drift will lead to elevated frequencies of derived alleles in an unfolded site frequency spectrum (Bustamante et al. 2001), where the ancestral allele is the variant most likely present in the ancestral population from which the expansion originated (Peter & Slatkin 2013). Given the deep divergence within this species between our focal populations and those in the Appalachian Mountains (Barnard‐Kubow et al. 2015), the ancestral allele identity was determined by the most common nucleotide across six individuals from three Appalachian populations (MD5, VA73, and WV72; see Barnard‐Kubow et al. 2015 for locations).

**Figure 1:**
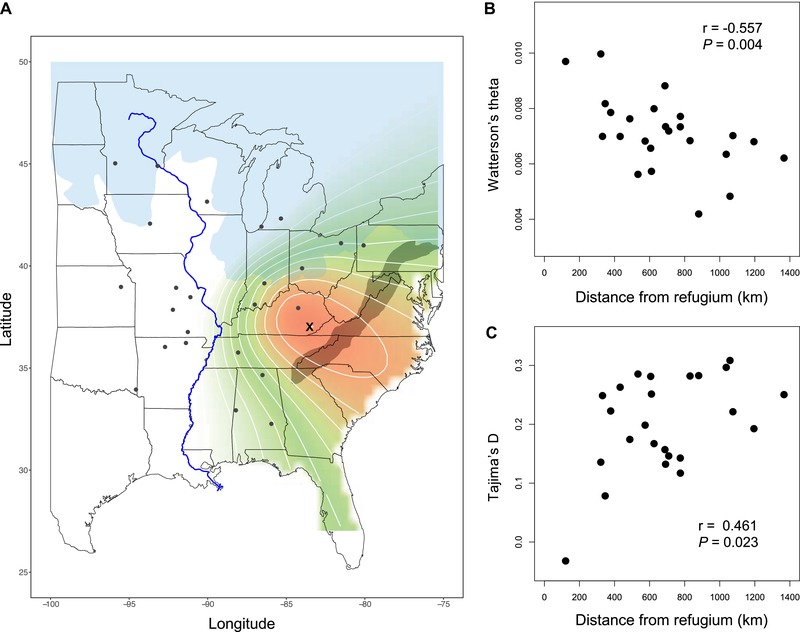
The impact of expansion from a glacial refugium on genetic diversity in *Campanula americana*. (A) The most likely origin of a geographic range expansion in *C. americana*. This position (X) was identified given a linear increase in the frequency of derived alleles with increasing distance from this refugium. Increasingly warmer colors indicate more likely localities of refugia. The extent of glaciers during the last glacial maximum is shown in light blue and the Appalachian Mountains are shaded in gray. (B) Watterson's theta ($\theta_{W})$ declines and (C) Tajima's *D* increases with distance from the refugium. The location of the 24 focal populations for which population genomic and ecological genetic metrics (genetic drift load and inbreeding depression) were measured are indicated by black points in panel A.

We analyzed spatial patterns in the site frequency spectrum of populations to identify the most likely refugium as the origin point of a geographic range expansion. This approach borrows a framework where the origin of a signal emanating from an unknown point is inferred by studying its differential receipt at points across the landscape (the “Time Difference of Arrival” or TDoA method; Gustafsson & Gunnarsson 2003). Given an assumed point of origin on a landscape, geographic distances between populations and this point (*d_i_*, *d_j_*) were calculated. Because genetic drift intensifies in regions far from the refugium, pairwise differences in these distances (*d*_ij_ = *d_i_* − *d_j_*) should be linearly related to pairwise differences in the frequency of derived alleles (ψ_ij_; Peter & Slatkin 2013). To identify the most likely origin, separate linear regressions were conducted between ψ_ij_ and d_ij_ using a set of possible origin points separated by 0.001 decimal degrees. These points spanned the range of 24 populations and 5^o^ beyond the minimal and maximal bounds of latitude and longitude. The most likely point of origin was identified from the regression with the largest coefficient of determination (*R* ^2^) and a positive slope coefficient. We repeated these analyses using two plants from the Atlantic Coast (VA71) or the Gulf Coast (FL16) to polarize alleles as ancestral (see Barnard‐Kubow et al. 2015 for locations), and this had little influence on the geographic location of the inferred refugium (Fig. 1A, Fig. S1).

### GENETIC DRIFT LOAD AND INBREEDING DEPRESSION

#### Crossing design

We collected single fruits from up to 40 maternal plants in each of the 24 focal populations in August 2015 (Table S1). We sowed seeds from each of 25 maternal families per population in metromix and turface (3:1). Seeds germinated in a growth chamber at 21°C/14°C (day/night) with 12‐hour day lengths. After 30 days, seedlings were vernalized in a cold room at 5°C for 45 days. In February 2016, a single seedling from each maternal family was transplanted into a cone‐tainer and placed in a greenhouse with 16‐hour day lengths. Flowering and fruit set spanned April to July.

Maternal plants from each of 24 populations were each subject to hand pollinations to create three cross types: (1) self‐fertilization (hereafter, “self”), (2) outcross fertilization by a donor from the same population (hereafter “within”), and (3) outcross fertilization by a donor from a different population (hereafter, “between”). The paternal plant for the within‐population cross was chosen randomly. For the between‐population crosses, each maternal plant of a given population was crossed with a randomly selected donor from a single population at a roughly similar latitude (Table S1). Between‐population crosses were not reciprocal. Thus, we generated 24 between‐population lines. Prior to pollinations, recipient flowers were emasculated. A single male‐phase flower was used to coat the recipient stigma with pollen. Across the three cross types, 1426 flowers from 482 individuals were pollinated (14–21 plants/population, mean = 20.1 plants/population) and upon ripening, we collected fruits.

#### Fitness of the F_1_ generation

From fruit produced by hand pollination, that is, a seed family (*n* = 1414), we sowed five seeds in each of two cells (*n* = 10/family) across 18 seedling trays in a randomized fashion (*n* = 14,140 seeds). F_1_ seeds were germinated in the same conditions as the parental generation. We scored the number of seedlings 26 days after sowing in each cell. Proportion germination was quantified as the number of seedlings that germinated divided by the number of seeds planted for each family. We then thinned each cell to a single seedling. Seedlings were vernalized in the same manner as their parents.

We randomly selected one seedling from each family (maternal plant × cross type) to transplant into the greenhouse. If there were fewer than 16 plants in a cross treatment within a population, we planted a second replicate of some families to achieve the goal of 20 plants per cross type. In total, we collected fitness data on 1424 F_1_ plants (mean = 19.8/cross type/population). For each plant, we scored survival to flowering, the occurrence of bolting, flower production, seed set, and above‐ground biomass. All plants that died prior to flowering and those that did not bolt within three months of transplanting were recorded. We monitored plants three times per week to record the date of first flower. We scored the number of open flowers on each plant once per week for four consecutive weeks beginning one week after initiation of flowering. On each plant produced through selfing and within‐population outcrossing, we applied outcross pollen from a single donor from the same population and cross type to an emasculated flower. For example, all plants from population IN77 in the self were crossed by a pollen donor from IN77 also in the self‐treatment. The resultant seed set thus reflects both maternal and paternal quality of a given population and cross type combination. When mature, we collected the hand‐pollinated fruits and counted seeds produced. We then harvested and dried the above‐ground tissue and measured the dry weight of each plant in grams on a Metler Toledo AG245 balance. Seed production of F_1_s was not scored for the between‐population hybrids because it could reflect F_2_ hybrid breakdown due to negative epistatic interactions rather than heterosis (Johansen‐Morris & Latta 2006).

Death was exceedingly rare (<1% of all F_1_s), but failure to bolt was more common (8.7%). Because reproduction in *C. americana* depends on bolting and subsequent flowering, we grouped survival and bolting into a single metric. To measure the proportion of plants that reached reproductive maturity (hereafter, “survival”), we summed the number of plants that died and did not bolt in each population and cross type combination, and divided this by the number of seedlings transplanted into the greenhouse. We estimated weekly flower production by averaging the sum of the four flower counts. In some cases (4.7% of plants), averages were based on fewer than four counts. Seed set was scored as the number of seeds produced. If a fruit did not set (5%), seed set was scored as zero.

#### Statistical analyses

To assess whether populations near the inferred refugium harbored the most genetic variation, we used the inferred origin of the range expansion based on the site frequency spectrum (Fig. 1A) and calculated the Haversine distance to each focal population. To connect the magnitude of genetic drift to the likely distance over which colonization events have occurred, we correlated distance from the refugium with population‐level genetic diversity ($\theta_{W}$ and Tajima's *D*).

We tested for drift load, the reduction in fitness resulting from genetic drift (Whitlock et al. 2000), and whether it varied among populations by comparing fitness of F_1_ progeny produced through within and between‐population crosses. We modeled germination rate, survival, flower production, and biomass using linear models with population, cross type, and their interaction as fixed effects. For proportion germination, we used a beta distribution; for survival, a binary distribution, and for flower production and biomass a normal distribution. Residuals for each model were normally distributed. In the analysis of germination, chamber was included as a random effect because seedlings were germinated in one of three growth chambers. A significant population × cross type interaction indicated that genetic drift load varied among populations. To evaluate the degree of inbreeding depression, we compared fitness of F_1_ progeny produced through within population outcrossing and selfing. We used the same models and distributions described for drift load. A significant population × cross type interaction indicated that populations varied in the magnitude of inbreeding depression.

To estimate population‐level drift load and inbreeding depression, we calculated the average values for each life stage for each cross type in each population. We then calculated cumulative fitness by multiplying population‐level germination proportion, survival proportion, flower production, and seed set (for inbreeding depression only). We calculated drift load at each life stage for each population using the equation (*W*_B_ − *W*_W_)/*W*_W_, where *W*_W_ is mean value of the within treatment and *W*_B_ is mean value of the between treatment. This estimates the proportional increase (heterosis) or decrease (outbreeding depression) in performance when crossing between populations relative to within populations. Using between‐population values in the denominator (e.g., Paland & Schmid 2003) yielded the same results. To calculate inbreeding depression, we used (*W*_W_ – *W*_s_)/*W*_W_ when *W*_W_ > *W*_s_ and (*W*_s_ – *W*_W_)/*W*_s_ when *W*_s_ > *W*_W_, where *W*_s_ is mean of the self‐treatment (Ågren & Schemske 1993).

To assess the effects of range expansion on components of the genetic load, we correlated drift load and inbreeding depression with geographic distance from the inferred refugium using Pearson product‐moment correlation. We then assessed the joint effects of distance from the origin, drift load, and inbreeding depression on variation in autonomous fruit set using a multiple linear regression. To assess the relative effects of each predictor, we standardized them using the “lm.beta” function in R.

Finally, to confirm spatial patterns in components of genetic load and autonomous fruit set were more strongly structured by range expansion than environmental conditions, we correlated each with the mean temperature of the growing season for each population (Worldclim, Bio 10, mean temperature of warmest quarter).

## Results

The spatial pattern of derived alleles in the site frequency spectra of *C. americana* populations is consistent with expansion from a refugium in eastern Kentucky, in the foothills of the Appalachian Mountains (37.021°N, 83.509° W, Fig. 1A). This inference follows from a tight relationship (*R* ^2^ = 0.420) between pairwise differences in site frequency spectra (ψ_ij_) and pairwise geographic distances between populations (*d*_ij_), in a model with this most likely point of origin. Most of the models we tested that had high coefficients of determination placed the refugium in the southern Appalachian Mountains, and populations near this refugium have the largest effective population sizes (Table 1; Fig. 1; Fig. S1).

Estimates of population genomic diversity (θ) varied from 0.004 to 0.011 across *C. americana* populations (Table 1). Watterson's theta ($\theta_{W}$) was strongly negatively associated with distance from the glacial refugium (*r* = −0.557; *P* = 0.004; Fig. 1B). Tajima's *D* was slightly negative (*D* = −0.032) in the population closest to the refugium, and significantly increased in populations farther from this putative point of origin (*r* = 0.461; *P* = 0.02; Fig. 1C). Observed declines in heterozygosity measured by the inbreeding coefficient (*F*) were not significantly associated with autonomous selfing ability at the population level (*r* = −0.345; *P* = 0.095).

**Figure 2:**
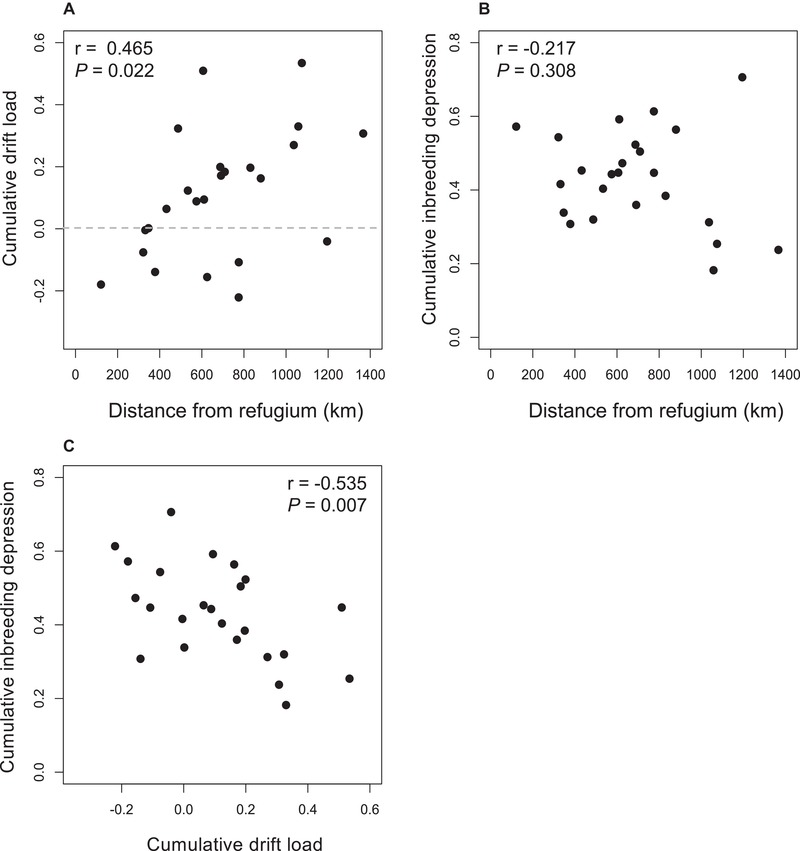
Range expansion shapes fixed genetic load but not segregating genetic load. (A) Cumulative drift load and (B) inbreeding depression plotted against the distance from the glacial refugium of *Campanula americana*. Points above the gray dotted line in (A) indicate populations that displayed heterosis, and those below it indicate populations displaying outbreeding depression. (C) Populations with elevated genetic drift load also have reduced inbreeding depression.

Across populations, heterosis was expressed in between‐population crosses for flower production and plant biomass, and its magnitude for each trait varied among populations (Table 2A). However, drift load was not detected for the early‐life traits of germination and survival (Table 2A). Drift load for flower production (*r* = 0.745, *P* < 0.001) and biomass (*r* = 0.437, *P* = 0.03) was elevated in populations farther from the refugium but at other life stages did not display a geographic pattern (Fig. S2). Cumulative drift load was on average low (0.11), but varied substantially among populations from modest outbreeding depression (−0.22) to heterosis (0.53) (Fig. 2A). Cumulative drift load increased with distance from the refugium (*r* = 0.465, *P* = 0.02; Fig. 2A). Likewise, the phenotypic expression of drift load was tightly associated with genomic signatures of drift. Drift load was positively correlated with Tajima's *D* (*r* = 0.538, *P* = 0.007) and negatively correlated with $\theta_{W}$ (*r* = −0.458, *P* = 0.025).

**Table 2:** Evaluation of genetic drift load and inbreeding depression on traits throughout the life cycle in 24 *Campanula americana* populations using self, within‐, and between‐population crosses

Source	Num DF	Germination	Survival	Flower production	Biomass	Seed production
**(A) Drift load**						
Population	23	1.26	0.34	5.20***	8.86***	–
Cross type	1	2.97	0	11.86**	6.85**	–
Pop. × cross type	23	1.10	0.42	3.17***	2.04**	–
Den Df		264	906	837	884	–
**(B) Inbreeding depression**						
Population	23	1.63*	2.00**	8.49***	9.72***	4.89***
Cross type	1	0.54	8.24**	11.24**	30.25***	62.17***
Pop. × cross type	23	1.01	0.60	0.75	1.03	0.68
Den Df		388	900	770	819	755

F_1_ fitness from within‐population outcrossing and between‐population outcrossing was compared to test for drift load (A), whereas fitness from self‐fertilization and within‐population outcrossing was compared to test for inbreeding depression (B). A significant cross type effect indicates the expression of drift load or inbreeding depression, while a significant interaction effect indicates populations differ in the magnitude drift load or inbreeding depression.

Inbreeding depression was expressed for all traits measured except germination (Table 2B). However, the magnitude of inbreeding depression did not differ among populations for any trait (Table 2B; Fig. S3) and was uncorrelated with distance from the refugium (Fig. S3). Cumulative inbreeding depression was 0.44 on average, and varied among populations from 0.18 to 0.70 (Fig. 2B). Although cumulative inbreeding depression was reduced in populations with a higher drift load (*r* = −0.54, *P* < 0.01, Fig. 2C), it was uncorrelated with distance from the refugium (*r* = −0.22, P = 0.31; Fig. 2B). Inbreeding depression was also uncorrelated with $\theta_{W}$ (*r* = 0.22, *P* = 0.31).

**Figure 3:**
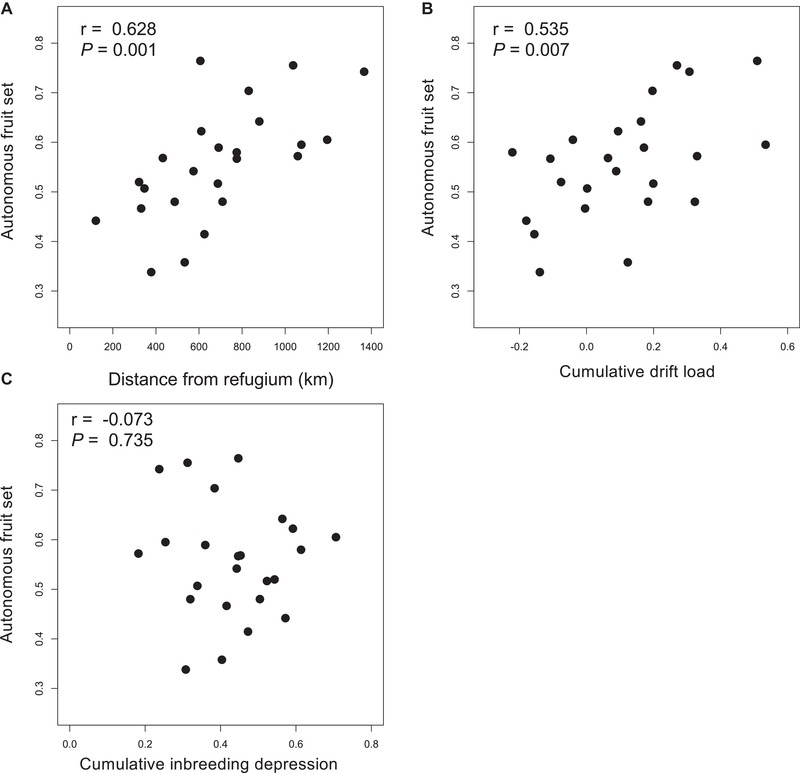
The ability to self‐fertilize is shaped by range expansion. The proportion of autonomous fruit set in *Campanula americana* populations increases with distance from the glacial refugium (A) and drift load (B), but it is not correlated with inbreeding depression (C). See Table 3 for relative effects of each predictor on autonomous fruit set.

Together, distance from the refugium and components of drift load explained 52% of the variation in autonomous fruit set (*P* = 0.0016), however only refugial distance and drift load were significant predictors, each having roughly similar magnitude of effect on autonomous fruit set (Table 3). The ability to autonomously self‐fertilize was greater in populations farther from the refugium (*r* = 0.63, *P* = 0.001; Fig. 3A), and that have a higher drift load (*r* = 0.54, *P* = 0.007; Fig. 3B). Cumulative inbreeding depression and autonomous fruit set, however, were unassociated (*r* = −0.07, *P* = 0.74; Fig. 3C).

**Table 3:** Results from a multiple linear model testing the effects of distance from the refugium, drift load, and inbreeding depression on variation in autonomous fruit set among populations. Parameter estimates are standardized for direct comparison

Parameter	Standardized estimate	*T*	*P*
Distance from refugium	0.472	2.71	0.014
Drift load	0.464	2.30	0.032
Inbreeding depression	0.278	1.52	0.144

Overall model: *R* ^2^ = 0.52, *F*_3,20_ = 7.35, *P* = 0.0017.

Mean growing season temperature was not correlated with drift load, inbreeding depression, or autonomous fruit set (Fig. S4).

## Discussion

Geographic patterns in population genomic and ecological genetic parameters in *C. americana* strongly suggest that postglacial range expansion has coincidentally structured variation in the ability to self‐fertilize and the accumulation of mutations. Both of these evolutionary responses are strongly related to the degree of genetic drift that populations have experienced, suggesting enhanced selection on the mating system in populations with historically small sizes. Furthermore, this work provides an empirical example of colonization's role in shaping reproductive traits. Given that numerous taxa have expanded out of glacial refugia in the recent past, a history of small effective population size following expansion may generate similar geographic distributions of traits and genetic load in many temperate plant populations.

### RANGE EXPANSION AND SPATIAL POPULATION GENETIC VARIATION

Previous analyses of genetic variation and spatial distribution models in *C. americana* suggested potential refugia of three clades in the Gulf Coast, Atlantic Coast, and southern Appalachians (Barnard‐Kubow et al. 2015). Chloroplast and nuclear phylogenies support the monophyly of populations west of the Appalachian Mountains (Barnard‐Kubow et al. 2015), and we found that populations increasingly far from the southern Appalachians have site frequency spectra with elevated frequencies of derived alleles. Not surprisingly, estimates of population genomic diversity ($\theta_{W\;}$and $\theta_{\pi }$) in *C. americana* are negatively associated with distance from this refugium. Increasing values of Tajima's D with distance to the refugium further imply that small populations have lost rare alleles in this species, as would be expected following repeated population bottlenecks during range expansion (Tajima 1989; González‐Martínez et al. 2017; Willi et al. 2018).

A decline in genetic diversity with distance from refugial sites as a signature of postglacial colonization is consistent with patterns found in many other taxa (Hewitt 2004; O'Connell et al. 2008; Gugerli et al. 2009; Sandoval‐Castro et al. 2014; however see Petit 2003). In *C. americana*, declines in effective population size are relatively modest and of uniform magnitude across geographic space. Although some contemporary populations may have persisted throughout periods of Pleistocene glaciation, many near the northern range edge must have been founded after glacial recession (Fig. 1A; French & Millar 2013). Regardless, such gradual declines in genomic diversity from the southern Appalachian Mountains to range edges suggests relatively similar numbers of founding individuals were sampled as the expansion wave moved progressively farther from a refugium in this species (Willi et al. 2018), and is consistent with a largely open expanse of suitable habitat (in contrast to Pujol et al. 2009).

### DRIFT LOAD, INBREEDING DEPRESSION, AND THE ABILITY TO SELF‐FERTILIZE

Geographic patterns for drift load coincide with signatures of range expansion from a refugium near the Appalachian Mountains. Elevated heterosis farther from the refugium reflects the phenotypic expression of the expansion load (Peischl et al. 2013; Peischl & Excoffier 2015). Additionally, elevated heterosis revealed when crossing between populations near the leading range edge implies that during expansion, populations accumulated deleterious mutations at different sites in the genome randomly through genetic drift. The geographic patterns of drift load and autonomous fruit set are each clinal in nature, as was observed for geographic patterns in effective population size. Thus, geographic patterns in putatively neutral genome‐wide variation and phenotypic parameters jointly reflect a historical signature of range expansion. Although the accumulation of expansion load has been modeled extensively (Peischl et al. 2013; Peischl & Excoffier 2015) and revealed at the genetic level (Lohmueller 2014; González‐Martínez et al. 2017), our study joins only one other that empirically demonstrates its predicted geographic pattern of phenotypic expression (Willi et al. 2018).

In *C. americana*, there is a strong negative association between drift load and inbreeding depression. Such a relationship is expected to emerge if some fraction of the segregating load causing inbreeding depression is converted into drift load during population bottlenecks (Paland & Schmid 2003; Oakley & Winn 2012). This may be particularly likely in tetraploid populations, where deleterious mutations reach relatively high frequencies under a balance between mutation and selection (Ronfort 1999). In this study, the magnitude of inbreeding depression was neither associated with a population's effective size nor its distance from the refugium. Inbreeding depression, which is mainly caused by partially or fully recessive mutations (Charlesworth & Wills 2009), is expected to decline following a population's recovery from a bottleneck when homozygous mutations are purged by natural selection (Kirkpatrick & Jarne 2000; Glemin 2003; Balick et al. 2015). This expectation follows from theoretical work in diploid populations (Glemin 2003; Balick et al. 2015; Peischl & Excoffier 2015), yet the purging of recessive mutations is less effective in tetraploids (Ronfort 1999; Layman & Busch 2018). Moreover, contemporary levels of inbreeding depression may not reflect those immediately following bottlenecks, because such declines in inbreeding depression should be erased relatively quickly after populations rebound in size (Kirkpatrick & Jarne 2000; Balick et al. 2015; Layman & Busch 2018).

The ability to self without a pollinator (i.e., autonomously) is a key component of the mating system. Contemporary *C. americana* populations with greater autonomy modify their selfing rate in response to the pollen limitation, whereas those with lower autonomy do not (Leibman et al. 2018; Koski et al. 2019). Although the extent of pollen limitation during the Pleistocene is little known, plants at the leading edge of the geographic range expansion likely encountered few mates or pollinators. Selection on mating system modifiers that enhance selfing when populations are very small can occur regardless of the segregating mutation load causing inbreeding depression (Lande & Schemske 1985; Barrett et al. 2014). Enhanced selection for selfing in colonizing populations is bolstered by recent comparative studies of many species, including invasive taxa, where robust associations are found between the ability to self‐fertilize and colonization (Brandvain et al. 2013; Razanajatovo et al. 2016; Grossenbacher et al. 2017).

In contrast to these historical arguments, autonomy neither causes elevated homozygosity nor influences the outcrossing rate of contemporary *C. americana* populations (Koski et al. 2019). There is also little apparent geographic structure to mate or pollinator availability across the species' geographic range (Koski et al. 2017). Viewed together, these results indicate that variation in a key reproductive trait is not explained by the current environments in which that trait is expressed. Instead, results point to an explicitly historic process for a geographic cline in the ability to self‐fertilize. Although this cline is likely selectively neutral in contemporary environments, such a geographic pattern is in line with observations of reduced outcrossing at higher latitudes in a broad diversity of plants (Moeller et al. 2017). Repeated founding events associated with colonizing new environments promote the concordant evolution of selfing modifiers and genetic load during range expansion (Willi et al. 2018). Together, both elevated drift load and selfing potential at range margins could synergistically limit adaptive potential through reductions in genetic diversity (Pujol & Pannell 2008; González‐Martínez et al. 2017).

Associate Editor: S. Wright

## Supporting information


**Table S1**: *Campanula americana* populations used in genomic and mating system studies with latitude and longitude of origin, the donor population used for the between‐population treatment for the estimate of heterosis, drift load, inbreeding depression, and autonomous fruit set.
**Figure S1**: Inferred origin of focal populations using Atlantic Coast (A) and Gulf Coast (B) populations as ancestral to identify derived alleles.
**Figure S2**: Drift load for proportion germination (A), survival (B), flower production (C) and plant biomass (D) in 24 populations of *Campanula americana* spanning a range of distances from the inferred glacial refugium.
**Figure S3**: Inbreeding depression for proportion germination (A), survival (B), flower production (C), seed production (D), and plant biomass (E) in 24 populations of *Campanula americana* spanning a range of distance from the putative glacial refugium.
**Figure S4**: Drift load, inbreeding depression, and autonomous fruit set plotted against average growing season temperature (Worldclim, Bio 10).
