# Supplementary material for: Selfing ability and drift load evolve with range expansion
Source: Evol Lett. 2019 Aug 29;3(5):500–12. doi: 10.1002/evl3.136 (PMC6791181; doi:10.1002/evl3.136)

Supporting Information

Koski et al., Selfing ability and drift load evolve with range expansion

Table S1*: Campanula americana* populations used in genomic and mating system studies with latitude and longitude of origin, the donor population used for the between-population treatment for the estimate of heterosis, drift load, inbreeding depression, and autonomous fruit set.

Population	Latitude	Longitude	Distance from Refugium (km)	Population for 'between' cross	Drift Load	Inbreeding Depression	Autonomous Fruit Set
KY51	37.938	-84.246	121	MO115	-0.18	0.572	0.442
OH119	39.885	-83.997	322	KS60	-0.076	0.543	0.52
IN77	38.107	-87.007	331	MO57	-0.004	0.416	0.467
IN46	39.149	-86.399	347	MO49	0.002	0.338	0.507
ALBG	34.656	-86.517	378	OK61	-0.139	0.308	0.338
TN19	35.758	-88.069	432	MO116	0.064	0.453	0.568
OH64	41.115	-81.518	487	MN118	0.323	0.32	0.48
PA27	41.008	-80.083	534	IA10	0.123	0.404	0.358
AL2012	32.267	-85.951	574	AR125	0.088	0.443	0.542
MI127	41.923	-86.583	606	MN117	0.509	0.447	0.764
MI126	42.321	-85.342	611	WI128	0.094	0.592	0.622
AL79	32.929	-88.208	625	AR56	-0.156	0.473	0.415
MO49	38.471	-91.104	687	IN77	0.199	0.523	0.517
MO116	36.765	-91.267	691	AL79	0.171	0.359	0.589
AR56	36.227	-91.386	709	ALBG	0.184	0.504	0.48
MO57	37.854	-92.218	775	KY51	-0.221	0.613	0.58
MO115	38.93	-92.012	775	OH119	-0.108	0.447	0.567
AR125	36.033	-92.716	831	TN19	0.197	0.384	0.704
WI128	43.15	-90.045	880	MI127	0.162	0.564	0.642
IA10	42.073	-93.672	1037	MI126	0.27	0.312	0.755
OK61	33.946	-94.567	1058	AL2012	0.33	0.182	0.572
KS60	38.971	-95.521	1075	IN46	0.534	0.254	0.595
MN117	44.901	-93.192	1195	OH64	-0.041	0.706	0.605
MN118	45.026	-95.888	1366	PA27	0.307	0.237	0.742

Figure S1: Inferred origin of focal populations using Atlantic Coast (A) and Gulf Coast (B) populations as ancestral to identify derived alleles. This position (X) was identified given a linear increase in the frequency of derived alleles with increasing distance from the refugium. Increasingly warmer colors indicate more likely localities of refugia and the Appalachian Mountains are shaded in gray. The location of the 24 focal populations for which population genomic and ecological genetic metrics (genetic drift load and inbreeding depression) were measured are indicated by black points.

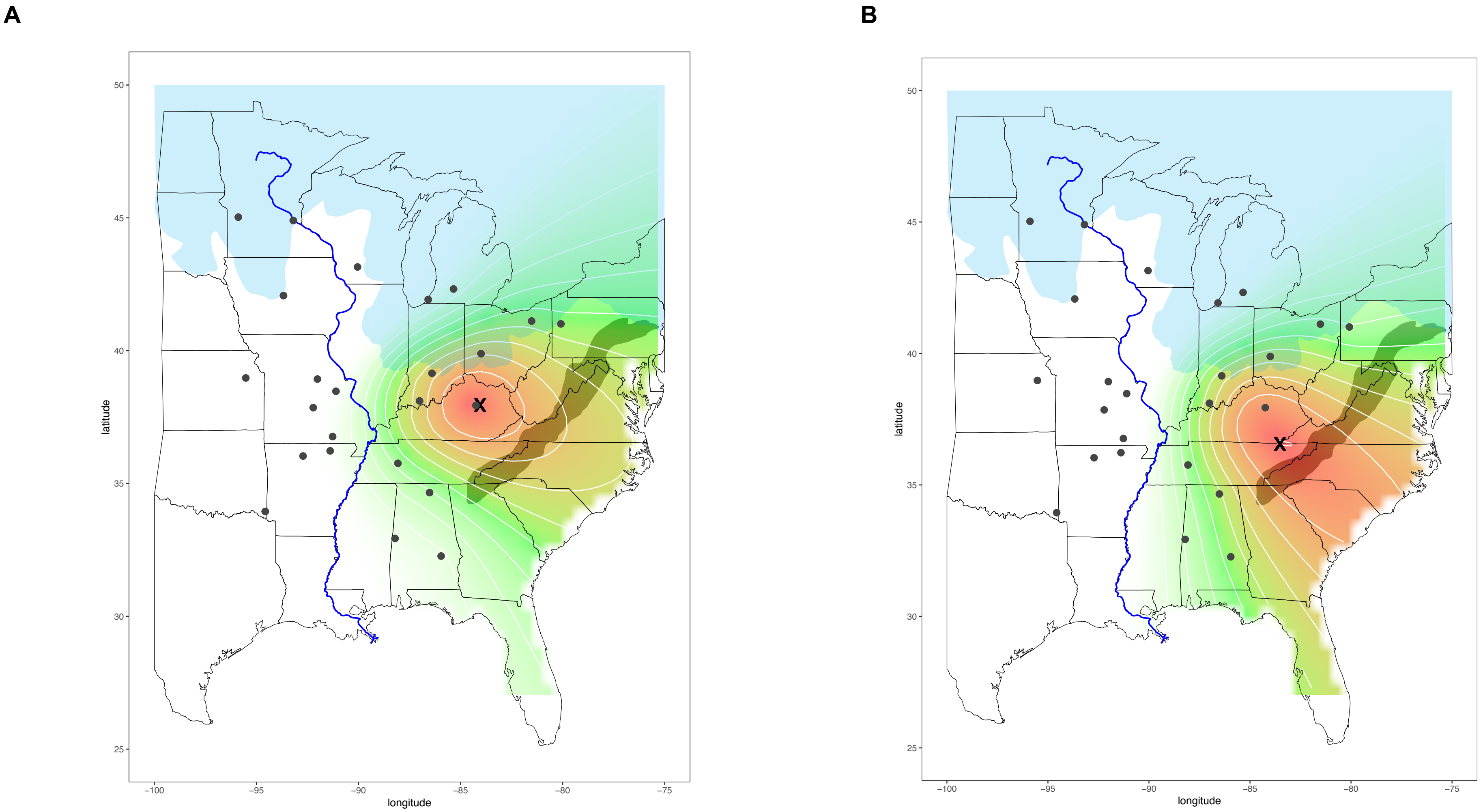

Figure S2: Drift load for proportion germination (A), survival (B), flower production (C) and plant biomass (D) in 24 populations of *Campanula americana* spanning a range of distances from the inferred glacial refugium. Statistics for significant relationships are shown.

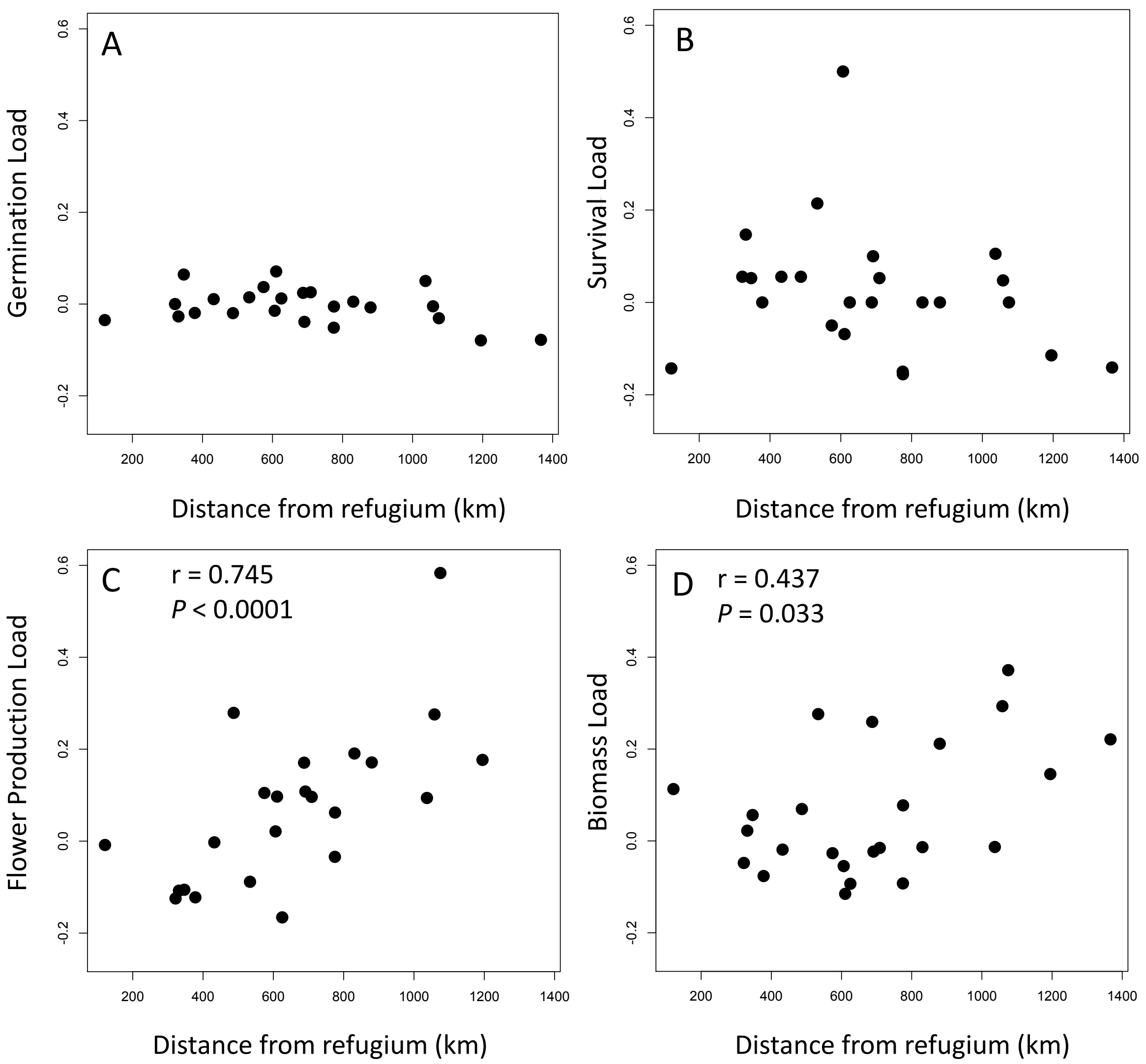

Figure S3: Inbreeding depression for proportion germination (A), survival (B), flower production (C), seed production (D), and plant biomass (E) in 24 populations of *Campanula americana* spanning a range of distance from the putative glacial refugium. None of the relationships are significant.

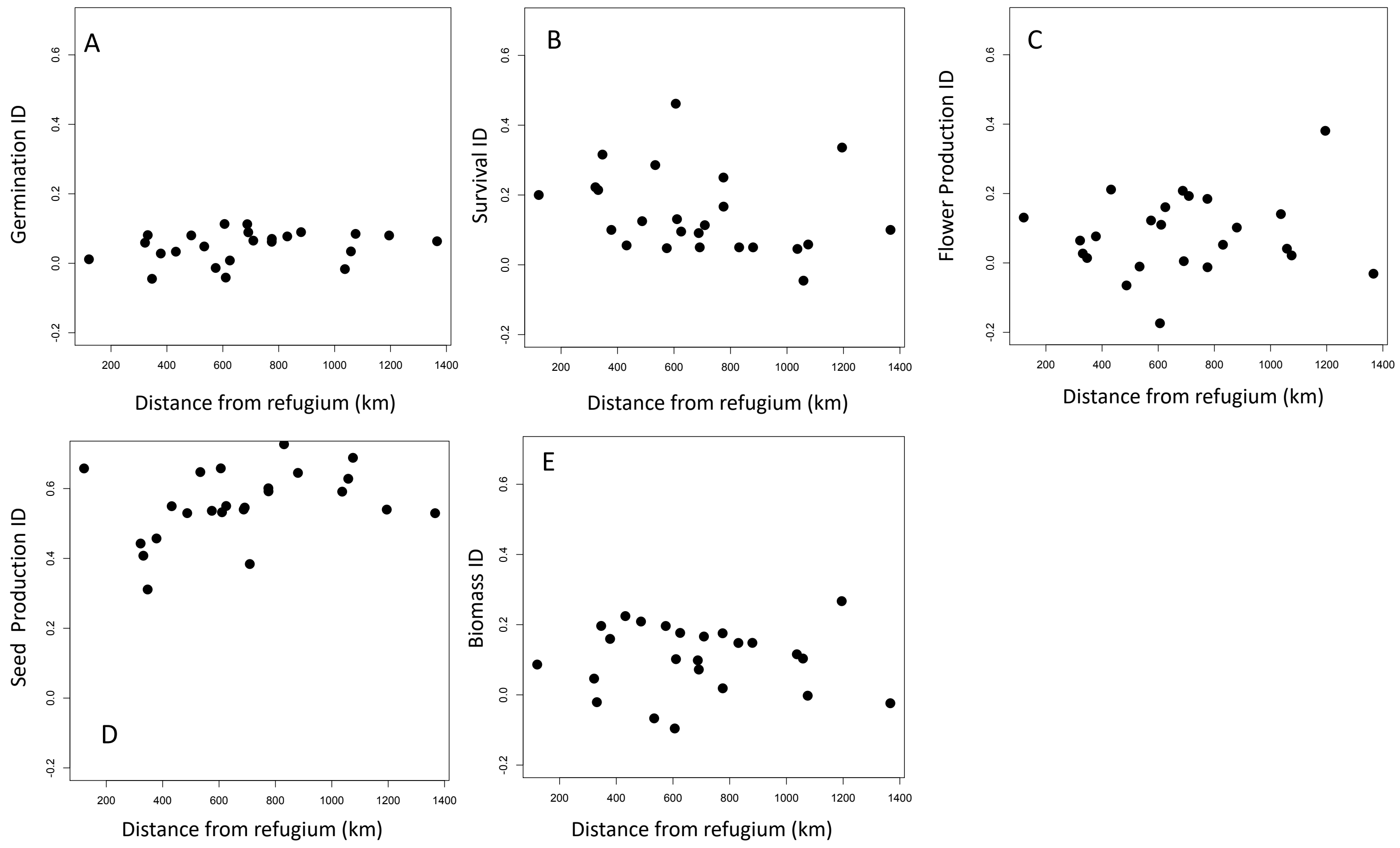

Figure S4: Drift load, inbreeding depression and autonomous fruit set plotted against average growing season temperature (Worldclim, Bio 10).

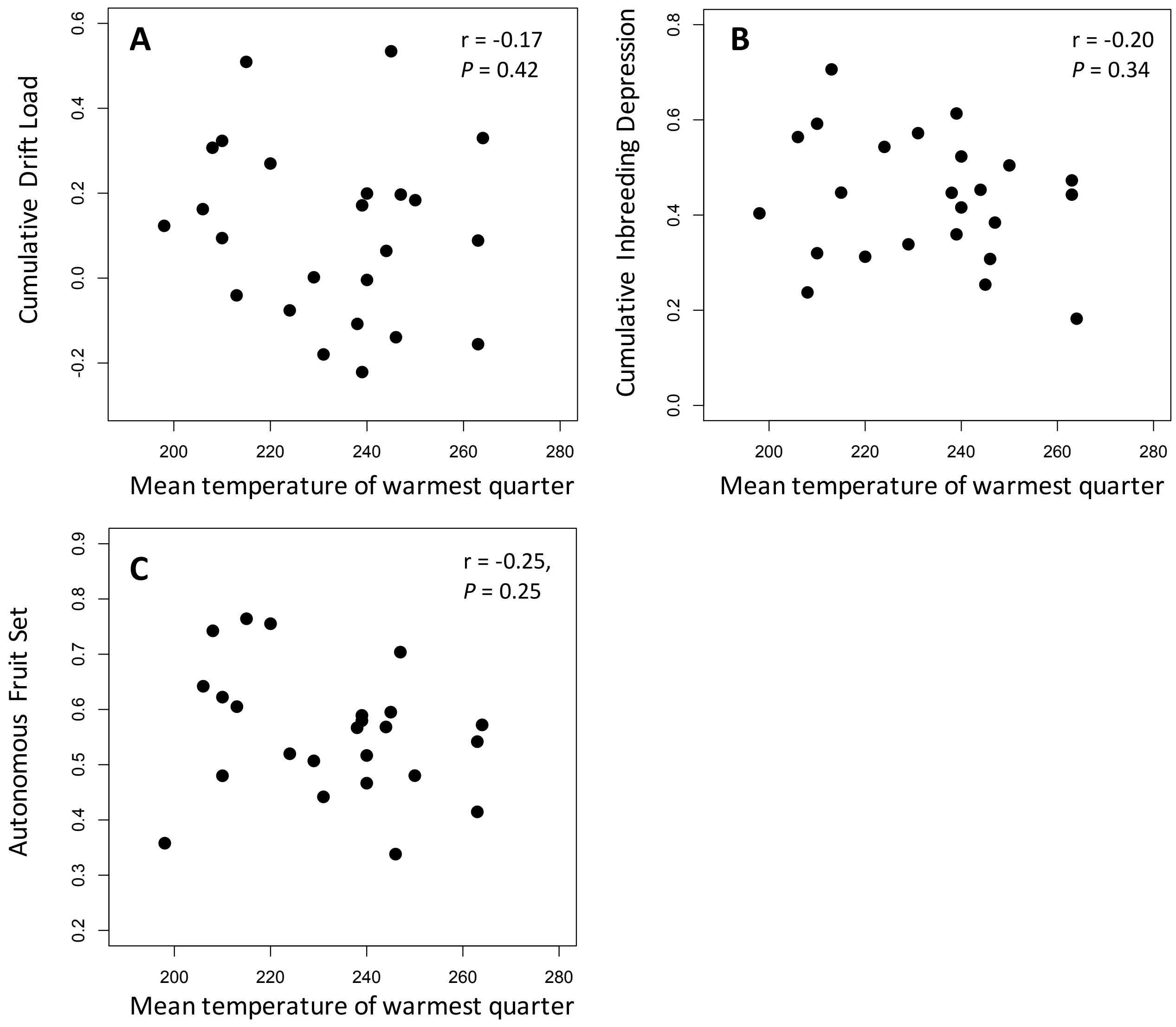

Supplement: Supplementary file 1 — Table S1: Campanula americana populations used in genomic and mating system studies with latitude and longitude of origin, the donor population used for the between‐population treatment for the estimate of heterosis, drift load, inbreeding depression, and autonomous fruit set. Figure S1: Inferred origin of focal populations using Atlantic Coast (A) and Gulf Coast (B) populations as ancestral to identify derived alleles. Figure S2: Drift load for proportion germination (A), survival (B), flower production (C) and plant biomass (D) in 24 populations of Campanula americana spanning a range of distances from the inferred glacial refugium. Figure S3: Inbreeding depression for proportion germination (A), survival (B), flower production (C), seed production (D), and plant biomass (E) in 24 populations of Campanula americana spanning a range of distance from the putative glacial refugium. Figure S4: Drift load, inbreeding depression, and autonomous fruit set plotted against average growing season temperature (Worldclim, Bio 10). [file EVL3-3-500-s001.pdf]
